# Genome-wide association study reveals new QTL and functional candidate genes for the small intestine length and cecum-colon length in Yorkshire pigs

**DOI:** 10.1093/jas/skaf085

**Published:** 2025-05-26

**Authors:** Liming Xu, Taoran Du, Qian Liu, Zhongyu Wang, Huilong Yang, Qingbo Zhao, Ruihua Huang, Pinghua Li

**Affiliations:** Key Laboratory of Pig Genetic Resources Evaluation and Utilization (Nanjing), Ministry of Agriculture and Rural Affairs, Institute of Swine Science, College of Animal Science and Technology, Nanjing Agricultural University, Nanjing, China; Key Laboratory of Pig Genetic Resources Evaluation and Utilization (Nanjing), Ministry of Agriculture and Rural Affairs, Institute of Swine Science, College of Animal Science and Technology, Nanjing Agricultural University, Nanjing, China; Key Laboratory of Pig Genetic Resources Evaluation and Utilization (Nanjing), Ministry of Agriculture and Rural Affairs, Institute of Swine Science, College of Animal Science and Technology, Nanjing Agricultural University, Nanjing, China; Key Laboratory of Pig Genetic Resources Evaluation and Utilization (Nanjing), Ministry of Agriculture and Rural Affairs, Institute of Swine Science, College of Animal Science and Technology, Nanjing Agricultural University, Nanjing, China; Key Laboratory of Pig Genetic Resources Evaluation and Utilization (Nanjing), Ministry of Agriculture and Rural Affairs, Institute of Swine Science, College of Animal Science and Technology, Nanjing Agricultural University, Nanjing, China; Key Laboratory of Pig Genetic Resources Evaluation and Utilization (Nanjing), Ministry of Agriculture and Rural Affairs, Institute of Swine Science, College of Animal Science and Technology, Nanjing Agricultural University, Nanjing, China; Key Laboratory of Pig Genetic Resources Evaluation and Utilization (Nanjing), Ministry of Agriculture and Rural Affairs, Institute of Swine Science, College of Animal Science and Technology, Nanjing Agricultural University, Nanjing, China; Huaian Academy, Nanjing Agricultural University, Huaian, China; Key Laboratory of Pig Genetic Resources Evaluation and Utilization (Nanjing), Ministry of Agriculture and Rural Affairs, Institute of Swine Science, College of Animal Science and Technology, Nanjing Agricultural University, Nanjing, China; Huaian Academy, Nanjing Agricultural University, Huaian, China

**Keywords:** cecum-colon length, functional candidate gene, pig, quantitative trait locus, small intestine length

## Abstract

The lengths of a pig’s small intestine length (**SIL**) and cecum-colon length (**C-CIL**) are crucial for nutrient absorption and substantially affect feed utilization efficiency. Thus, selecting for increased intestinal lengths could enhance feed digestibility. However, conventional methods for measuring these traits pose considerable challenges. To address these drawbacks, we explored the use of molecular breeding techniques to refine the selection process. In this study, 413 age-matched Yorkshire barrows were humanely killed, and their SIL and C-CIL were measured. Genotyping was conducted using a 50K single nucleotide polymorphism (**SNP**) array, and the SNP-chip data were imputed to whole-genome sequencing data (**iWGS**). The heritability estimates for SIL and C-CIL were 0.25 and 0.26, respectively. Utilizing genome-wide association studies based on chip and iWGS data, significant SNPs on *Sus scrofa chromosomes* (**SSC**) 2 and SSC9 for SIL traits and SSC16 for C-CIL traits were identified. Specifically, two novel quantitative trait loci (**QTLs**) regulating SIL on SSC2 and SSC9 were identified. Furthermore, one novel QTL regulating C-CIL on SSC16 was identified. Bayes fine mapping narrowed the confidence intervals for these QTLs to 1.91 Mb (from 42.37 to 44.28 Mb on SSC2) and 1.92 Mb (from 43.96 to 45.92 Mb on SSC9) for SIL and 1.96 Mb (from 11.14 to 13.10 Mb on SSC16) for C-CIL. Based on the biological functions of genes, *SRY-box transcription factor 6* on SSC2 and *apolipoprotein A4*, *SID1 transmembrane famil*y *member 2*, *transgelin*, and *transmembrane serine protease 13* on SSC9 were identified as novel candidate genes for SIL. Likewise, *cadherin 10* on SSC16 was identified as the novel candidate gene for C-CIL. Overall, these findings offer insights into identifying the causal genes and mutations affecting SIL and C-CIL. Additionally, they highlight novel molecular markers that can be utilized for the molecular breeding of intestine traits in Yorkshire pigs.

## Teaser

Identifying genetic markers for small intestine and cecum-colon length lays the foundation for improved feed digestibility in Yorkshire pigs.

## Introduction

The expanding global population has heightened the competition for food resources between humans and livestock, posing a formidable challenge to sustainable agricultural practices (Herrero et al. 2023). The increasing human demand for meat has contributed considerably to this concern. Pork production represents approximately one-third of global meat output; it generates a substantial annual requirement for grain resources to sustain this level of production (Gozalo-Marcilla et al., 2021). Thus, enhancing the feed utilization efficiency in pigs to achieve cost savings and production efficiency has become a critical issue.

The intestine is central to the digestive system, responsible for digesting and absorbing a wide spectrum of nutrients (Spiller, 1994; Gao et al., 2010). Gut development directly affects major economic traits in livestock, such as meat and milk production (Bach et al., 2017). In pigs, the intestine is primarily divided into the small and large intestines. The length of the small intestine length (**SIL**) is significantly correlated with the body length, which directly influences meat yield (Hounnou et al., 2002; Bekheit et al., 2020). A longer small intestine enables the digesta to remain inside for a prolonged period, enabling more digestion and absorption (Fish et al., 2024). Consequently, it facilitates greater nutrient absorption, thus enhancing feed utilization efficiency and promoting growth and development. Therefore, the length of the small intestine is strongly associated with daily weight gain (Barea et al., 2011). The cecum and colon serve as the key sites for fiber digestion in pigs (Drochner, 1993; Wilfart et al., 2007). In the cecum and colon, microorganisms secrete carbohydrate-active enzymes that break down fiber into monosaccharides, which are later absorbed by the body (Bhattacharya et al., 2015; Xu et al., 2021). Short-chain fatty acids—produced during dietary fiber fermentation by gut microbes in the large intestine—provide 7% to 17% of the energy required for growing pigs (Dierick et al., 1989; Anguita et al., 2006). A longer cecum and colon establish a more expansive habitat for microorganisms, thereby enhancing fiber digestion capacity (Zhang et al., 2022).

Genetic factors are central to the growth and development of the intestine. Genes, such as *keratinocyte growth factor*, *epidermal growth factor*, and *growth hormone*, are associated with the development of the small intestine. However, only *insulin-like growth factor 1* and *lactoferrin* have been identified as the key genes directly affecting its length (Steeb et al., 1994; Lindemans et al., 2015; Yang et al., 2017; Jensen et al., 2022). A quantitative trait locus (**QTL**) on porcine chromosome 4 significantly affecting the length of the small intestine has been identified at the genomic level (Andersson et al., 1994). Additionally, 13 QTL regions have been identified to affect the intestinal length in chickens (Gao et al., 2009). Furthermore, multiple QTL regions affecting the traits of the duodenum, jejunum, ileum, and overall SIL in chickens have been identified (Mignon-Grasteau et al., 2015). However, research on QTLs affecting the lengths of the small and large intestines in pigs is limited. Moreover, the existing genes fail to explain the phenotypic variation in these lengths, necessitating research on additional QTLs and causal genes that affect these traits in pigs.

The Yorkshire pig is one of the most extensively bred lean meat breeds globally. In this study, we aimed to identify novel QTLs and candidate genes affecting the lengths of the small intestine and cecum-colon (C-CIL) in Yorkshire pigs, providing a theoretical foundation for selecting pigs with enhanced feed utilization efficiency.

## Materials and Methods

### Ethics statement

All experimental protocols involving animals were approved by the Nanjing Agricultural University Animal Care and Use Committee (Certification No.: SYXK (Su) 2022-0031).

### Animal population and data collection

A total of 413 Yorkshire barrows, all raised on the same farm, were included in this study. The pigs were of comparable ages and were fed a similar diet and fasted for 1 d in the lairage before being humanely killed in five batches at Xuzhou Zhengda Food Co., Ltd. The carcass weight of each pig was recorded, and ear tissue samples were collected for subsequent DNA extraction. The average carcass weight was 99.8 (± 0.6) kg. The entire intestine was removed from the abdominal cavity, with the mesentery dissected away. The intestinal tract was divided into the small and large intestines at the ileocecal junction. Each section was laid flat on a level surface. The lengths of the small intestine—extending from the anterior end of the duodenum to the terminal end of the ileum—and cecum-colon—extending from the anterior end of the cecum to the posterior end of the colon—were measured using a soft tape measure. Fourteen and four values were missing for the SIL and C-CIL, respectively.

### Genotyping and quality control

Genomic DNA was extracted from ear tissue samples using the Megi General Nucleic Acid Extraction Kit, with DNA integrity confirmed by 0.8% agarose gel electrophoresis. DNA quantification and quality assessment were conducted using a NanoDrop 2000 spectrophotometer, with the criteria set at an OD260/280 ratio of 1.7 to 2.1 and a concentration >50 ng/μL. Genotyping was conducted using the Compass Pig 50K Plus Breeding BeadChip (Tianjin, China), including 57,466 single nucleotide polymorphisms (**SNPs**). The physical positions of these SNPs were updated to the Sus Scrofa 11.1 build of the pig reference genome (Sscrofa11.1). Quality control was conducted using PLINK v1.9 software (Fabbri et al., 2020). Animals with >10% missing genotypes were excluded. SNPs with a call rate <0.90 and a minor allele frequency (MAF) <0.05 were discarded. After quality control, 36,582 high-quality SNPs from 413 animals were retained for further analysis.

### Genetic parameters estimation

Heritability was calculated using the AI-REML algorithm within the single-trait model of the HIBLUP software (Yin et al., 2023). The model is presented in equation 1:


$$\begin{array}{l} y=Xa+Zg+e, \end{array}$$ (1)

where ***y*** is the phenotype vector, ***a*** is the fixed effects vector (including the carcass weight and slaughter batch), ***g*** is the random additive effect vector, ***e*** is the random residual effect vector, and ***X*** and ***Z*** are the incidence matrices. Random effects were assumed to follow a normal distribution and be independent of each other. We assumed that ***g***~***N*** (0, **G**$\sigma_{A}^{2}$) and ***e***~***N*** (0, ***I***$\sigma_{e}^{2}$), where ***G*** is the additive genetic relationship matrix based on SNP markers, ***I*** is the identity matrix, $\sigma_{A}^{2}$ is the additive genetic variance, and $\sigma_{e}^{2}\;$ is the residual variance. Phenotypic variance ($\sigma_{p}^{2}$) was defined as the sum of $\sigma_{A}^{2}$ and $\sigma_{e}^{2}$. Heritability (h2) was calculated as the ratio of $\sigma_{A}^{2}$ and $\sigma_{p}^{2}$.

### Imputation

Whole-genome resequencing data from 1,662 pigs served as the reference panel. It comprised resequencing data from 1,602 pigs across multiple breeds obtained from the PigGTEX project and data from 60 Suhuai pigs (Qiu et al., 2024; Teng et al., 2024). The 50K chip data obtained from sequencing were imputed to the resequencing level using the default parameters of Beagle software. SNPs with a dosage *R*-squared <0.9 and MAF <0.05 were removed. After quality control, data from 413 animals with 5,752,897 SNPs were retained for further analysis.

### Genome-wide association analysis

The genome-wide association analysis (**GWAS**) was conducted using the LDAK software (Speed et al., 2012). The mixed linear model is described below:


$$\begin{array}{l} y=Xa+Wb+k+e \end{array}$$ (2)

where ***y*** is the phenotype vector, ***a*** is the fixed effects vector (including the carcass weight and slaughter batch), ***b*** is the substitution effect of the alleles, ***k*** is the random polygenic effect after ***N*** (0, ***K***$\sigma_{A}^{2}$) distribution, ***K*** is the genomic kinship matrix calculated using the LDAK-Thin algorithm under the default parameters, ***e*** is the random residual effect vector, and ***X*** and ***W*** are the incidence matrices (Wang et al., 2021).

In conventional models, a candidate SNP in both fixed and random effects can lead to overfitting and reduced statistical power. Nonetheless, it is impractical to simultaneously consider a candidate SNP for both effects because of substantial computational expenses. Thus, the “leave-one-chromosome-out” approach was adopted (Yang et al., 2014). Using the Bonferroni correction, a significance threshold of 1/*N* was established, where *N* is the total number of filtered SNPs (36,582) from SNP-chip data. For imputed to whole-genome sequence (**iWGS**) data, a genome-wide significance threshold of *N* based on the effective SNP count (142,973) was established. This count was determined using the PLINK v1.9 software with the parameter “--indep-pairwise 50 5 0.2” (Qiu et al., 2024). The proportion of phenotypic variance explained by SNP additive effects was calculated as follows: 2*p*(1 − *p*)*β*^2^/$\sigma_{P}^{2}$, where *p* is the MAF of the SNP, *β* is the estimated allele substitution effect, and $\sigma_{P}^{2}$ is the phenotypic variance. Finally, Manhattan and quantile–quantile (**Q-Q**) plots were generated utilizing the CMplot R package (Yin et al., 2021).

### Bayes fine mapping

To compile a comprehensive list of potential genes within the QTLs of interest, the Bayesian framework CAVIARBF was utilized. It helped to delineate the confidence intervals (**CIs**) of QTLs using iWGS data (Chen et al., 2015). For each significant signal, SNPs attaining statistical significance (*P* < 0.01) within a 2 Mb window centered on the lead SNP (± 1.0 Mb) were determined as potential variant sets (Maller et al., 2012). Using the CAVIARBF v0.2.1 software, marginal association statistics and linkage disequilibrium (LD) patterns were analyzed. This approach facilitated identifying the minimal set of variants comprising causal variants with a posterior probability of 95%. The identified QTLs were compared with data from the Pig QTL database (https://www.animalgenome.org/) to identify novel QTLs.

### Function of candidate genes

Insights into the functional roles of candidate genes were derived from extensive literature searches in PubMed (https://pubmed.ncbi.nlm.nih.gov/) and methodical queries in the GeneCards online database (https://www.genecards.org/). Additionally, a Phenome-Wide Association Study (PheWAS) of the candidate genes was conducted using the PigBiobank database (https://pigbiobank.farmgtex.org/).

## Results

### Descriptive statistics and heritability estimates

In the Yorkshire pig population, the SIL and C-CIL ranged from 17.68 to 30.52 m and 4.41 to 7.24 m, respectively. The average SIL was 24.07 m (SE = 0.10), and the average C-CIL was 5.65 m (SE = 0.03). The coefficient of variation (**CV**) was 8.52% for SIL and 8.99% for C-CIL.

Table 1 summarizes the heritability values for SIL and C-CIL and the phenotypic and genetic correlation coefficients. The estimated heritability values for SIL and C-CIL were 0.25 and 0.26, respectively, indicating both traits are influenced by genetic factors.

**Table 1.** Descriptive statistics and genetic parameters for the SIL and cecum-colon length (C-CIL)

Traits	N^1^	Min^2^	Max^3^	Mean^4^±SE^5^	CV^6^(%)	*h* ^2^±SE^7^	*P*-value
SIL, m C-CIL, m	399 409	17.68 4.41	30.52 7.24	24.07 ± 0.10 5.65 ± 0.03	8.52 8.99	0.25 ± 0.09 0.26 ± 0.08	0.026 0.002

^1^Number of individuals with phenotypic records.

^2^Minimum of phenotype.

^3^Maximum of phenotype.

^4^Mean of phenotype.

^5^Standard error.

^6^Coefficient of variation.

^7^Heritability of trait.

### Genome-wide association studies

**Figure 1.**
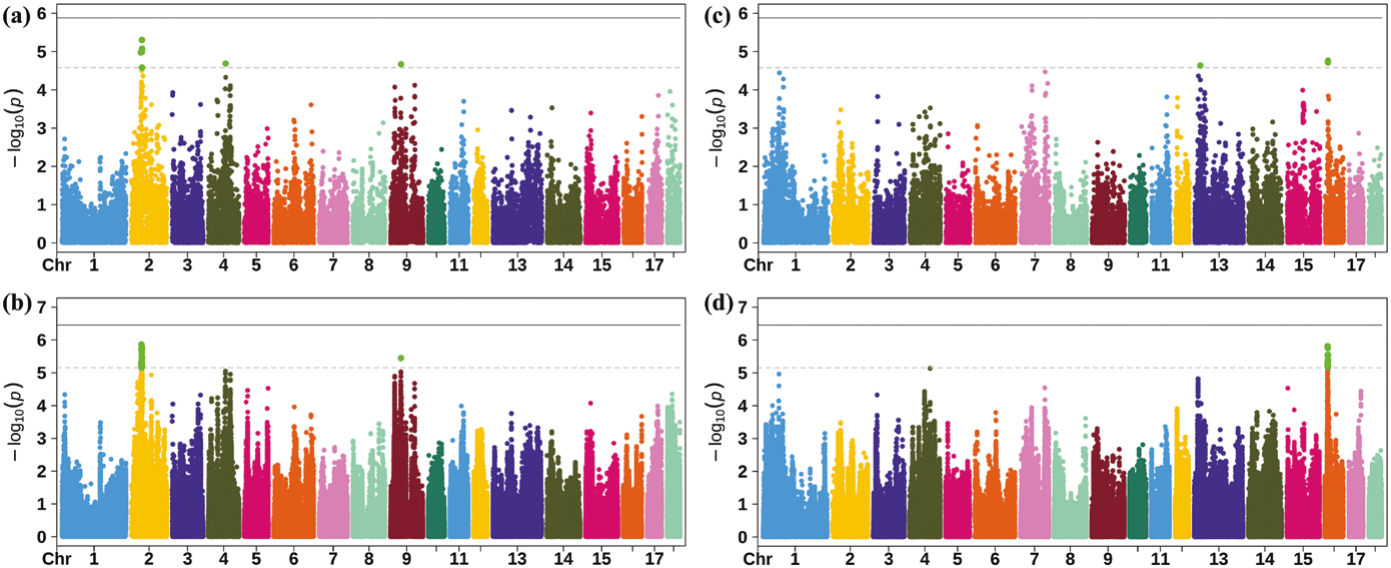
Manhattan plots of GWAS for the SIL and cecum-colon length (C-CIL) traits. (a) and (c) were based on chip data, and (b) and (d) were based on imputed whole-genome sequence data. Negative log10 *P*-values of SNPs (y-axis) were plotted against their corresponding genomic positions (x-axis). The horizontal solid and dashed lines represent the genome-wide significance and suggestive thresholds, respectively. The green dots between the solid and dashed lines represent SNPs with *P*-values below the suggestive threshold but above the genome-wide significance threshold.

Principal component analysis results did not indicate population stratification (Supplementary Figure S1). To assess the accuracy of the inferred genotypes, 5% of SNPs were randomly removed and re-imputed into the SNP-chip dataset. The allele concordance rate and correlation of these SNPs were used to evaluate the imputation accuracy, with average values of 98.46% and 98.67%, respectively. GWAS was conducted using both chip and iWGS data for SIL and C-CIL. Figure 1 illustrates Manhattan plots for the traits, and Supplementary Figure S2 illustrates the corresponding Q-Q plots.

Based on SNP-chip data, 13 SNPs distributed across *Sus scrofa chromosomes* (**SSC**) 2, SSC4, and SSC9 were significantly associated with SIL. rs80947993 explained 4.73% of the phenotypic variance. The most significant SNPs on SSC4 and SSC9 explained 4.85% and 4.60% of the phenotypic variance, respectively (Table 2). The λ-value of GWAS was 1.167. Upon using iWGS data, 367 SNPs on SSC2, SSC4, and SSC9 were significantly associated with SIL. rs1112713048 explained 5.37% of the phenotypic variance. In contrast, the most significant SNP on SSC9 explained 5.48% of the phenotypic variance (Table 2). The λ-value of GWAS was 1.2.

**Table 2.** GWAS results for the SIL and cecum-colon length (C-CIL) based on chip and imputed to whole-genome sequence (iWGS) data

Trait	Data set	Chromosome^1^	Position	Top SNP	*P*-value of the top SNP^2^	Variance^3^	Nearest gene to the top SNP
SIL	Chip data	2	45347961	rs80947993	4.93 × 10^−6^	4.73%	*SPON1*
		4	73972428	rs80923720	2.04 × 10^−5^	4.85%	*TOX20*
		9	44558610	rs81410456	2.14 × 10^−5^	4.60%	*SIDT2*
	iWGS data	2	43279549	rs1112713048	1.37 × 10^−6^	5.37%	*ENSSSCG00000052631*
		9	44922083	rs327674164	3.56 × 10^−6^	5.48%	*DSCAML1*
C-CIL	Chip data	13	23600960	rs346314104	2.30 × 10^−5^	3.98%	*SCN11A*
		16	12161386	rs81464561	1.72 × 10^−5^	4.64%	*CDH10*
	iWGS data	16	12103514	rs340114120	1.54 × 10^−6^	5.03%	*CDH10*

^1^ *Sus scrofa chromosome*, the same as below.

^2^ *P*-value according to the Wald test.

^3^Phenotypic variation explained by the top SNP.

For C-CIL, four SNPs distributed across SSC13 and SSC16 were significantly associated with the trait. rs81464561 explained 3.98% of the phenotypic variance. In contrast, the most significant SNP on SSC13 explained 4.64% of the phenotypic variance (Table 2). The λ-value for GWAS was 1.189. Upon using iWGS data, 528 SNPs on SSC16 were significantly associated with C-CIL. rs340114120 explained 5.03% of the phenotypic variance (Table 2). The λ-value of GWAS was 1.197.

### Bayes fine mapping

**Figure 2.**
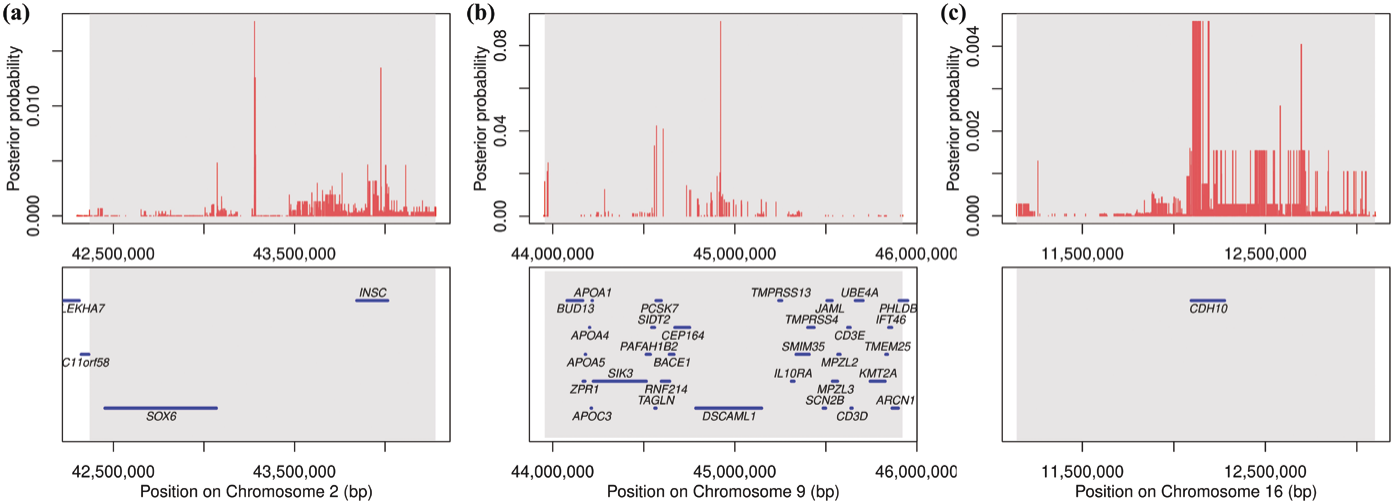
Bayes fine mapping of the QTL for SIL on chromosomes 2 (a) and 9 (b) and for cecum-colon length on chromosome 16 (c). The x-axis represents the physical positions of SNPs in the genome, and the y-axis indicates posterior probability. Vertical red lines represent SNPs, and blue lines represent genes. The gray rectangle indicates the 95% CI of QTL.

For each signal, the capacity of each variant to explain the detected effect within a 2Mb window (±1.0 Mb relative to the lead SNP) was assessed. Consequently, the minimal ensemble of variants, encompassing the causal variant with 95% certainty, was identified. BFM using iWGS data was conducted to identify the QTL affecting both traits. For SIL, the 95% CIs of QTL ranged from 42.37 to 44.28 Mb on SSC2 (Figure 2a) and 43.96 to 45.92 Mb on SSC9 (Figure 2b). For C-CIL, the 95% CIs of QTL ranged from 11.14 to 13.10 Mb on SSC16 (Figure 2c). A comparison with the Pig QTL Database suggested that two QTL (SSC2: 42.37 to 44.28 Mb and SSC9: 43.96 to 45.92 Mb) affecting SIL and one QTL (SSC16: 11.14 to 13.10 Mb) affecting C-CIL were the novel discoveries.

### Candidate genes

**Figure 3.**
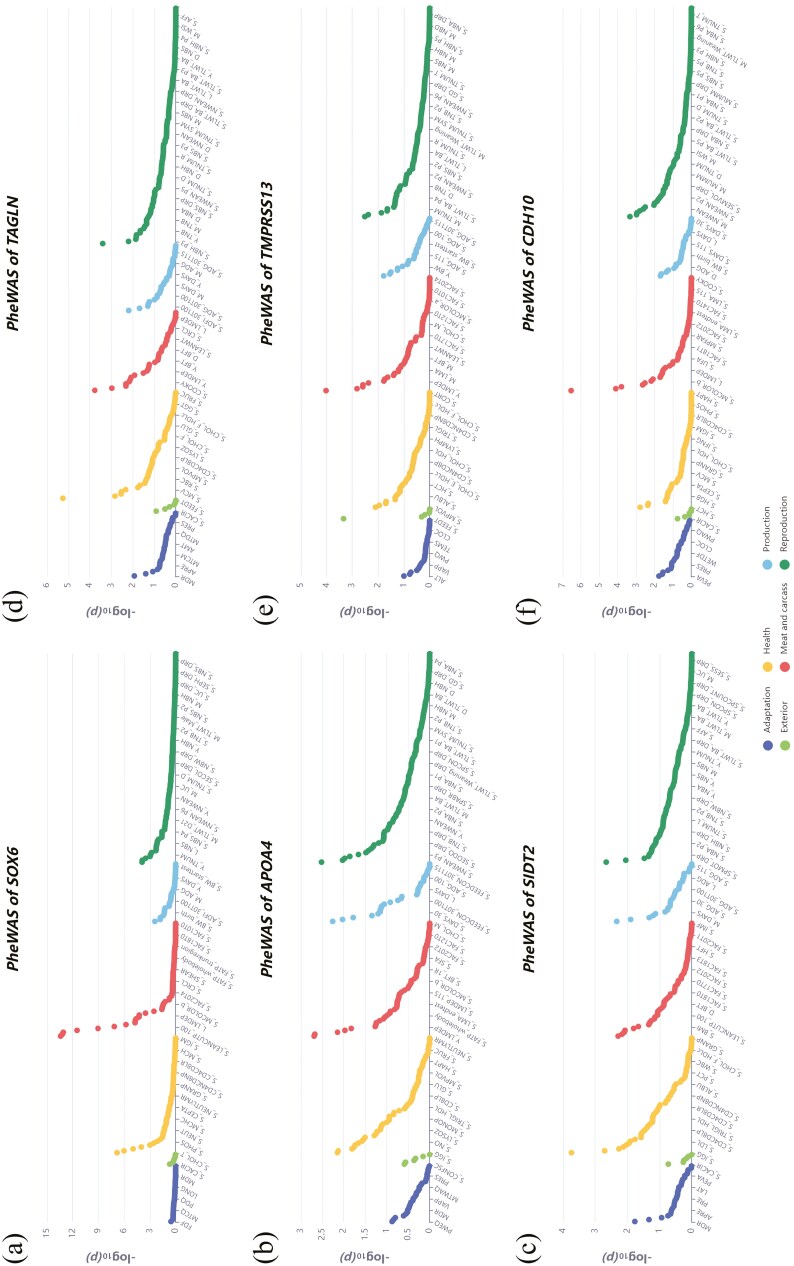
The results of candidate gene PheWAS. Panels (a), (b), (c), (d), (e), and (f) represent the PheWAS results for *SOX6*, *APOA4*, *SIDT2*, *TAGLN*, *TNPRSS13*, and *CDH10*, respectively.

Table 3 lists 31 protein-coding genes within the QTL regions influencing SIL and C-CIL. Based on reported biological functions, *SRY-box transcription factor 6 (SOX6)*, *apolipoprotein A4 (APOA4)*, *SID1 transmembrane famil*y *member 2 (SIDT2)*, *transgelin (TAGLN)*, and *transmembrane serine protease 13 (TMPRSS13)* were identified as the candidate genes associated for SIL. Meanwhile, *cadherin 10 (CDH10)* was identified as the candidate gene for C-CIL. A PheWAS analysis was conducted using the PigBiobank database (Figure 3). PheWAS results indicated significant associations between these genes and major production traits, including daily weight gain, average daily feed intake, and body weight (Table 4).

**Table 3.** Protein-coding genes for the SIL and cecum-colon length (C-CIL) in the QTL regions identified through Bayes fine mapping

Trait	Chromosome	QTL region (Mb)	Genes^1^
SIL	SSC2 SSC9	42.37–44.28 43.96–45.92	*INSC* ***SOX6*** *C11orf58* *APOA1 BUD13* ***APOA4*** *APOA5 SIK3 ZPR1 APOC3 PCSK7* ***SIDT2*** *CEP164 PAFAH1B2 BACE1 RNF214* ***TAGLN*** *DSCAML1* ***TMPRSS13*** *UBE4A JAML PHLDB TMPRSS4 CD3E SMIM35 TMEM25 MPZL2 SCN2B ARCN1 CD3D*
C-CIL	SSC16	11.14–13.10	***CDH10***

^1^The genes indicated in bold were identified as potential functional candidate genes for the corresponding trait under analysis based on literature support and their known functional roles based on the GeneCards online database.

**Table 4.** Significantly associated traits in the candidate gene PheWAS production category results

Genes	Trait	Study symbol	*P*-value	Category^1^
*SOX6* *SOX6* *SOX6* *SOX6* *SOX6* *SOX6* *APOA4* *APOA4* *APOA4* *APOA4* *SIDT2* *SIDT2* *SIDT2* *TAGLN* *TAGLN* *TNPRSS13* *TNPRSS13* *TNPRSS13* *CDH10* *CDH10* *CDH10*	Average daily gain Body weight Days Average daily gain Average daily gain (30–115 kg) Body weight (birth) Days Days Body weight Average daily feed intake (30–100 kg) Days Body weight Average daily gain Body weight Days Average daily gain Body weight Body weight Average daily gain Average daily gain Average daily gain (30–100 kg)	D_ADG D_BW L_DAYS L_ADG S_ADG_30T115 S_BW_birth D_DAYS M_DAYS L_BW S_ADFI_30T100 D_DAYS L_BW D_ADG L_BW D_DAYS M_ADG M_BW D_BW Y_ADG M_ADG S_ADG_30T100	3.87 × 10^−3^ 1.58 × 10^−2^ 1.64 × 10^−2^ 3.56 × 10^−2^ 4.67 × 10^−2^ 4.81 × 10^−2^ 5.53 × 10^−3^ 9.47 × 10^−3^ 1.67 × 10^−2^ 4.56 × 10^−2^ 4.68 × 10^−3^ 1.26 × 10^−2^ 4.73 × 10^−2^ 6.52 × 10^−3^ 2.08 × 10^−2^ 1.66 × 10^−2^ 2.88 × 10^−2^ 3.15 × 10^−2^ 2.04 × 10^−2^ 2.27 × 10^−2^ 4.20 × 10^−2^	Growth Growth Growth Growth Growth Growth Growth Growth Growth Feed intake Growth Growth Growth Growth Growth Growth Growth Growth Growth Growth Growth

^1^The main categories corresponding to the traits.

## Discussion

Limited reports have elucidated the heritability of pig SIL and C-CIL. Previous studies have reported heritability estimates ranging from 0.30 to 0.37 for the intestinal length in chickens (Pan et al., 2024). In this study, heritability estimates for SIL and C-CIL were 0.25 and 0.26, respectively, classifying them as moderately heritable traits.

GWAS are widely used for genetic improvement in livestock (Goddard et al., 2016). In this study, SNP-chip data were used to preliminarily identify genetic variants associated with SIL and C-CIL in Yorkshire pigs. Bonferroni-corrected *P*-values were calculated to determine the SNP effects (Tong et al., 2023). The genome-wide significance threshold was set at 1.37 × 10^−6^ (0.05/36,582), whereas the suggestive significance threshold was set at 2.73 × 10^−5^ (1/36,582). For SIL, significant SNPs identified through chip-based GWAS were distributed across SSC2, SSC4, and SSC9. For C-CIL, significant SNPs were distributed across SSC13 and SSC16, with the most prominent signal detected on the *CDH10* gene.

Commercial SNP arrays for pigs include only a fraction of the common and low-frequency variants in the genome, restricting their efficacy in detecting the key variants influencing traits. WGS data provides a solution. However, the expense of sequencing a population is often prohibitive. To address this limitation, imputing higher-density or WGS data from low-density SNP arrays provides a cost-efficient alternative. Genotype imputation enhances the identification of causative variants or associated LD markers, aiding QTL detection and precise mapping (Van Son et al., 2019). Stringent Bonferroni-corrected thresholds were utilized for the iWGS analysis, estimating 142,973 SNPs using PLINK v1.9 with the “--indep-pairwise 50 5 0.2” parameter (Wang et al., 2021). Compared with chip-based GWAS, iWGS-based GWAS identified more significant SNPs associated with both SIL and C-CIL. Using chip-based GWAS, SNPs on SSC2 and SSC9 for SIL and those on SSC10 for C-CIL were identified within corresponding genomic regions using iWGS data. However, SNPs on SSC4 for SIL in chip-based GWAS were insignificant in iWGS-based GWAS. Similarly, SNPs on SSC13 for C-CIL in chip-based GWAS were insignificant in iWGS-based GWAS. This discrepancy may result from applying a more stringent threshold in iWGS-based GWAS.

Determining CIs using SNP-based GWAS is challenging because it assesses individual SNPs without considering LD variability among adjacent SNPs. To define the CIs more accurately, we conducted Bayes fine mapping with iWGS data. BFM integrates GWAS summary statistics with LD information, enhancing the efficiency of detecting QTLs and identifying causal mutations (Choquet et al., 2017). A comparison with the Pig QTL Database indicated two novel QTLs (SSC2: 42.37 to 44.28 Mb and SSC9: 43.96 to 45.92 Mb) influencing SIL and one novel QTL (SSC16: 11.14 to 13.10 Mb) influencing C-CIL.

All protein-coding genes within the QTL CIs were examined for functional information to identify the candidate genes for SIL and C-CIL. Based on the biological roles, *SOX6*, *APOA4*, *SIDT2*, *TAGLN*, and *TMPRSS13* have been identified as the candidates for SIL. Similarly, *CDH10* was identified as the candidate gene for C-CIL. Intestinal epithelial cells, lining the intestinal epithelium, undergo constant renewal and replacement to preserve gut functionality. During embryonic development and the early postnatal growth phase, rapid cell proliferation contributes to intestinal elongation. *SOX6* belongs to the *SOX* family and is central to cell differentiation and tissue development (An et al., 2011; Lefebvre, 2019). Researchers have highlighted the importance of *SOX6* in intestinal epithelial cell proliferation. For instance, *SOX6* is upregulated during intestinal development and is essential for epithelial cell differentiation (Kinchen et al., 2018). Despite limited studies linking *SOX6* to epithelial cell proliferation, its broader developmental role suggests its potential implications (Li et al., 2024). Therefore, *SOX6* may promote small intestine development by affecting intestinal epithelial cell proliferation. *APOA4* encodes an apolipoprotein primarily associated with lipid metabolism. Its expression and functionality may indirectly influence intestinal health and cell proliferation (Delgado-Lista et al., 2010). *SIDT2* is associated with transporting inorganic sulfate and sulfated compounds across cell membranes (Zhao et al., 2023). Sulfates can be absorbed as nutrient components, thereby promoting epithelial cell proliferation and differentiation. *TAGLN* is located within the QTL region spanning 43.96 to 45.92 Mb on SSC9; it regulates smooth muscle contraction (Tsuji-Tamura et al., 2021). *TMPRSS13* is involved in various biological processes, including cell signaling, cell migration, and tissue remodeling (Varela et al., 2020). It may indirectly influence intestinal development by modulating the interactions between epithelial cells and the extracellular matrix and by regulating the integrity and permeability of the intestinal epithelial barrier. For C-CIL, *CDH10* spans 11.14 to 13.10 Mb on SSC16. It acts as a calcium-dependent cell adhesion molecule and is central to forming tight junctions and establishing cell polarity, which are vital for preserving tissue structure and function (Williams et al., 2005). *CDH10* facilitates intercellular adhesion by interacting with similar adhesion molecules on adjacent cells through its extracellular domain (An et al., 2015). Furthermore, *CDH10* may regulate intracellular signaling pathways affecting cell proliferation, migration, and differentiation (Li et al., 2015). By influencing adhesion properties and signaling pathways of epithelial cells, *CDH10* can indirectly influence intestinal health and development. Additionally, the PheWAS results suggested that these genes may indirectly affect production traits by influencing SIL and C-CIL. Specifically, the findings indicated significant associations between these genes and major production traits, such as daily weight gain, average daily feed intake, and body weight. Therefore, the genetic control of intestinal length may have broader implications for growth efficiency and overall productivity in livestock.

## Conclusions

We identified two novel QTL (SSC2: 42.37 to 44.28Mb and SSC9: 43.96 to 45.92Mb) for SIL and one novel QTL (SSC16: 11.14 to 13.10Mb) for C-CIL in Yorkshire pigs. *SOX6*, *APOA4*, *SIDT2*, *TAGLN*, and *TMPRSS13* are considered functional candidate genes for SIL, while *CDH10* is regarded as a functional candidate gene for C-CIL. These findings showed the complexity of the genetic mechanisms of SIL and C-CIL, providing a valuable basis for subsequently pinpointing the causal genes and mutations.

## Supplementary Data

Supplementary data are available at *Journal of Animal Science* online.

skaf085_suppl_Supplementary_Figure_S1

skaf085_suppl_Supplementary_Figure_S2

## Data Availability

The phenotype data (https://doi.org/10.6084/m9.figshare.26877769.v1) SNP-chip data (https://doi.org/10.6084/m9.figshare.28794782.v1) used in this study are deposited in the figshare repository.

## Abbreviations:

C-CIL: cecum-colon length
CV: coefficient of variation
GWAS: genome-wide association studies
iWGS: imputed to whole-genome sequence
LD: linkage disequilibrium
Q-Q: quantile–quantile
QTL: quantitative trait locus
SIL: small intestine length
SNP: single nucleotide polymorphism
