# Supplementary figures and images for: Genome-wide association study reveals new QTL and functional candidate genes for the small intestine length and cecum-colon length in Yorkshire pigs
Source: J Anim Sci. 2025 May 26;103:skaf085. doi: 10.1093/jas/skaf085 (PMC12205944; doi:10.1093/jas/skaf085)

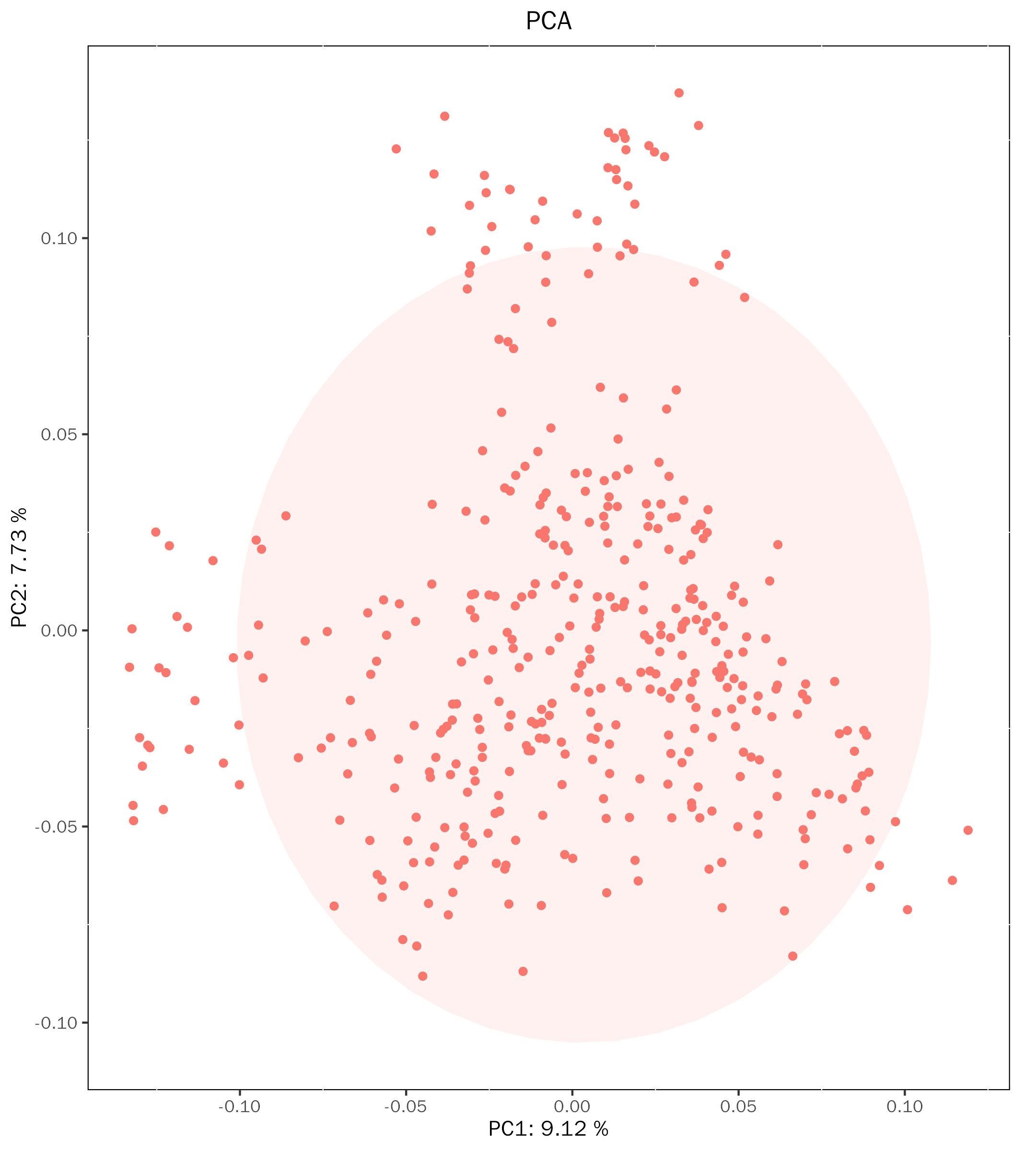

Supplement: skaf085_suppl_Supplementary_Figure_S1 [file skaf085_suppl_supplementary_figure_s1.jpeg]

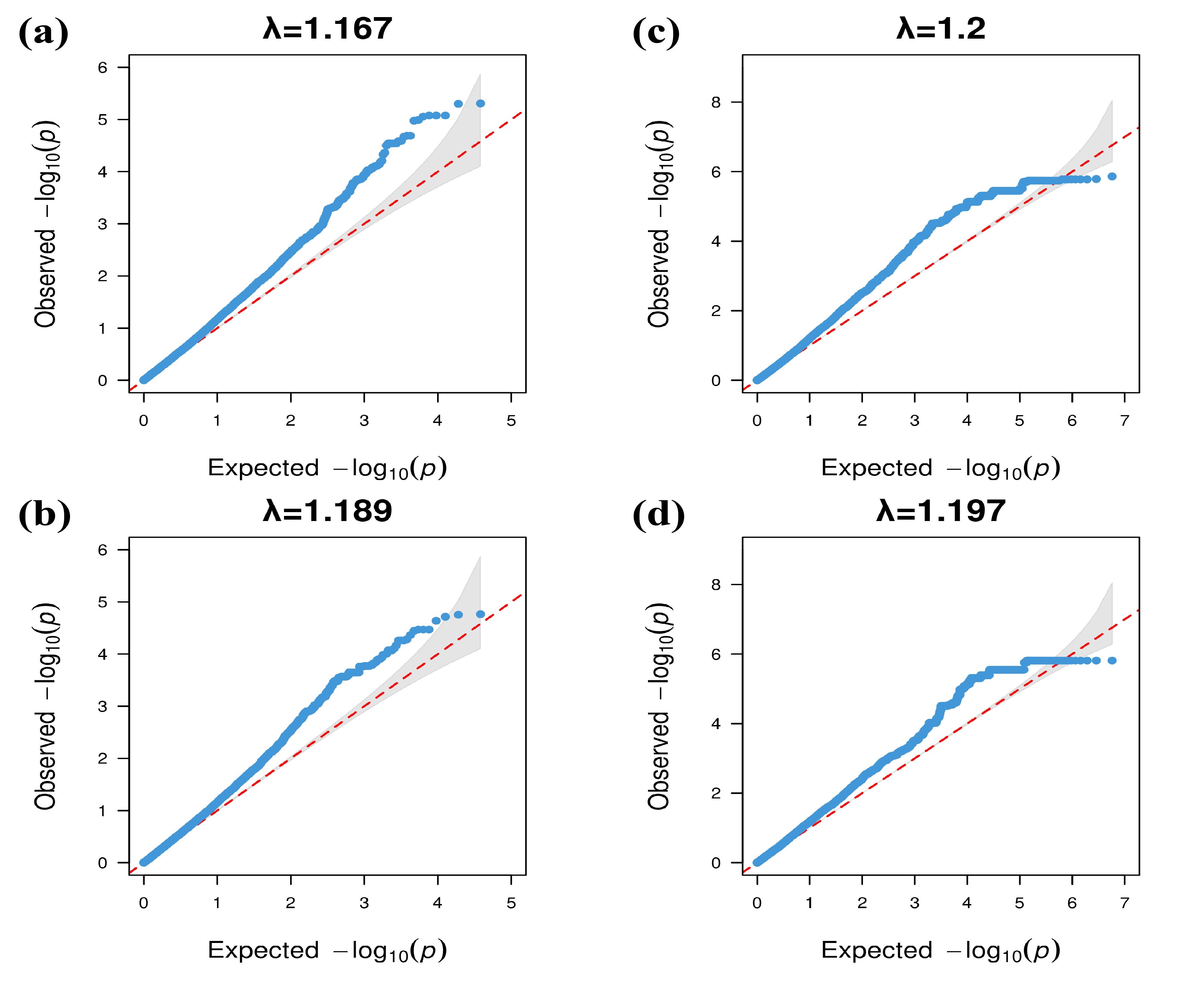

Supplement: skaf085_suppl_Supplementary_Figure_S2 [file skaf085_suppl_supplementary_figure_s2.jpeg]
